# Editor's Report

**Published:** 1949-04

**Authors:** 


### Editor's Report

**October, 1948**

THE Spring issue did not appear until May, since when we have been able to produce only one number : but " Autumn " will be in your hands in a few days, and " Winter " before the end of the year. We shall thus be able to return to the official plan with " Spring, 1949 " in January. One legacy of the war is a very great increase in the cost of typesetting and paper. This has imposed certain economies, for example, a limitation of the length of articles, omission of lists of past presidents, subscribers, and the less important examinations results, etc.

You will notice that our old title has been set out in a different style, to being back into prominence our claim to be the *Journal* for the West of England and South Wales. For another result of the war was a considerable loss of members and also of non-member subscribers to the *Journal*, a loss which it is our next task to replace. We compiled a list of former Bristol students, a tedious business. A complimentary copy of the *Journal*, with explanatory letter, has been sent to all former students (338), to practitioners in various parts of the Region (272) and to the secretaries of local medical societies (58), in all 668 copies. Already subscriptions are coming in, and the secretaries have agreed to bring the matter before their societies this Autumn, from which we may expect more. Bath and Cardiff have appointed Local Correspondents, and we hope that soon we shall again, as formerly, be able to give some account of meetings and activities throughout the South West. And with an increased circulation we shall be able to provide a larger and better *Journal*. This must necessarily depend largely on the increase in non-member subscriptions, for obviously the space devoted to the affairs of any area must bear some relationship to the number of subscribers in that area.

The accounts for the year show that the *Journal* made a very small profit during the year, after allowing for the expense of the complimentary issue. For the information of members the expenses for three issues have been " dissected " into components, demonstrating that typesetting accounts for more than half the total expense of each issue. The price of books reviewed in the *Journal* and handed over to the library during the year is £24 6s. 6d.; seventy-eight journals were received in exchange priced at £100 : this £124 6s. 6d. represents " invisible " revenue which the *Journal* earns for the Society.

The Accounts and Balance Sheet require a little explanation : partly because members' subscriptions have been shown as " grants " to the *Journal*, creating the impression that these amounts were subsidies additional to the actual subscription. During the war the Society did not meet and the accounts were not audited. In 1946 the accounts for seven years were audited together. Unfortunately there were two important omissions : first, the auditors took account of sums received from the Society (£635 0s. 0d.), but treated these as the total amount due, whereas, in fact, the subscriptions due on behalf of members amounted to £819 18s. 0d. The accounts published in the *Journal* (1946, p. 156) showed the balance due as an asset ; and consequently increased the credit balance by this amount.

| | £ | s. | d. | | £ | s. | d. |
|---|---|---|---|---|---|---|---|
| Credit Balance : | | | | Subscriptions due (7 years) | 819 | 18 | 0 |
| Published .. .. .. .. | 192 | 12 | 11 | *Less* amount paid .. .. | 635 | 0 | 0 |
| Auditors' figure .. .. | 7 | 14 | 11 | | | | |
| | £184 | 18 | 0 | | £184 | 18 | 0 |

The second omission was due to accounts for printing the 1946 issues not being available to the auditors. The small credit balance shown above should really have been a large deficit :

| | £ | s. | d. | | £ | s. | d. |
|---|---|---|---|---|---|---|---|
| Spring, 1946, cost .. .. | 114 | 5 | 7 | Credit Balance (Auditors' figure) .. .. .. .. .. | 7 | 14 | 11 |
| Summer and Autumn .. | 138 | 14 | 10 | " True " Debit Balance, ignoring the sum of £184 18s. 0d. due from Society | 326 | 3 | 5 |
| Winter .. .. .. .. .. | 80 | 17 | 11 | | | | |
| Cost of 1946 issue .. .. | £333 | 18 | 4 | | £333 | 18 | 4 |

### JOURNAL ACCOUNTS, 1947

**EXPENDITURE**

| | £ | s. | d. | £ | s. | d. |
|---|---|---|---|---|---|---|
| Printing— | | | | | | |
| Spring and Summer | 150 | 9 | 9 | | | |
| Autumn .. .. .. | 81 | 10 | 6 | | | |
| Winter .. .. .. | 61 | 0 | 10 | | | |
| | | | | 293 | 1 | 1 |
| Auditors' Fee .. .. | | | | 2 | 2 | 0 |
| | | | | 295 | 3 | 1 |
| Surplus for year .. | | | | 56 | 5 | 10 |
| | | | | £351 | 8 | 11 |

**INCOME**

| | £ | s. | d. |
|---|---|---|---|
| Non-members' Subscriptions .. | 22 | 15 | 0 |
| Members' Subscriptions .. .. .. | 244 | 0 | 0 |
| Arrears (1945) paid on Account.. | 84 | 13 | 11 |
| | £351 | 8 | 11 |

**LIABILITIES**

| | £ | s. | d. |
|---|---|---|---|
| J. W. Arrowsmith Ltd., as above | 293 | 1 | 1 |
| Auditors* .. .. .. .. .. .. .. | 1 | 11 | 6 |
| | £294 | 12 | 7 |

**ASSETS**

| | £ | s. | d. | £ | s. | d. |
|---|---|---|---|---|---|---|
| Cash at Bank .. .. | | | | 24 | 15 | 0 |
| Balance, 1946 .. .. | 326 | 3 | 5 | | | |
| *Less* Surplus, 1947 .. | 56 | 5 | 10 | | | |
| Debit Balance, 1947 .. | | | | 269 | 17 | 7 |
| | | | | £294 | 12 | 7 |

* I cannot explain why amount due to Auditors is less than the fee charged under expenditure.—ED.

**INCOME AND EXPENDITURE ACCOUNT for the year ended 30th September, 1948**

| EXPENDITURE | £ | s. | d. | INCOME | £ | s. | d. |
|---|---|---|---|---|---|---|---|
| Printing— | | | | Members' Subscriptions .. .. | 356 | 0 | 0 |
| Spring, 1948 .. .. .. .. .. | 120 | 5 | 5 | Non-members' Subscriptions .. | 20 | 3 | 6 |
| Summer, 1948 .. .. .. .. | 57 | 11 | 9 | | | | |
| Autumn, 1948 .. .. .. .. .. | 60 | 7 | 4 | | | | |
| Winter, 1948 (estimate) .. .. | 50 | 0 | 0 | | | | |
| | 288 | 4 | 6 | | | | |
| Complimentary issue .. .. .. | 75 | 7 | 1 | | | | |
| Stationery (office) .. .. .. .. | 7 | 18 | 1 | | | | |
| Due to J. W. Arrowsmith Ltd .. | 371 | 9 | 8 | | | | |
| Auditors Fee .. .. .. .. .. .. | 3 | 3 | 0 | | | | |
| Postage .. .. .. .. .. .. .. | 1 | 10 | 0 | | | | |
| Surplus for year .. .. .. .. | | | 10 | | | | |
| | £376 | 3 | 6 | | £376 | 3 | 6 |

**BALANCE SHEET as at 30th September, 1948**

| LIABILITIES | £ | s. | d. | ASSETS | £ | s. | d. |
|---|---|---|---|---|---|---|---|
| Due to J. W. Arrowsmith Ltd. .. | 371 | 9 | 8 | Cash at Bank, current account .. | 34 | 19 | 0 |
| Auditors Fee .. .. .. .. .. .. | 3 | 3 | 0 | Due from Society for 1948 .. .. | 69 | 16 | 11 |
| | | | | Deficit— £ s. d. | | | |
| | | | | September, 1947 .. ..269 17 7 | | | |
| | | | | *Less* Surplus.. .. .. 10 | | | |
| | | | | | 269 | 16 | 9 |
| | £374 | 12 | 8 | | £374 | 12 | 8 |

**ANALYSIS OF JOURNAL EXPENSES, 1948**

| | *Summer* £ | s. | d. | *Autumn* £ | s. | d. | *Winter* £ | s. | d. |
|---|---|---|---|---|---|---|---|---|---|
| Original Articles .. .. .. | 38 | 13 | 4 | 40 | 2 | 8 | 35 | 6 | 0 |
| Reviews Notes, etc. .. .. .. | 20 | 16 | 4 | 18 | 1 | 6 | 22 | 3 | 10 |
| Advertisements .. .. .. | 16 | 8 | 4 | 16 | 1 | 5 | 14 | 16 | 1 |
| Typesetting .. .. .. .. .. | 40 | 2 | 2 | 40 | 9 | 11 | 42 | 15 | 9 |
| Paper .. .. .. .. .. | 4 | 4 | 8 | 4 | 8 | 8 | 4 | 8 | 5 |
| Printing, Cover, Despatch .. .. | 32 | 1 | 2 | 29 | 7 | 0 | 25 | 1 | 9 |
| | 76 | 8 | 0 | 74 | 5 | 7 | 72 | 5 | 11 |
| Received for Advertisements .. | 18 | 16 | 3 | 13 | 18 | 3 | 17 | 17 | 6 |
| Nett .. .. .. .. .. .. | £57 | 11 | 9 | £60 | 7 | 4 | £54 | 7 | 5 |

Although it is not formally audited, Mr. Keeler has agreed that this account is correct and accurately represents the position on 30th September, 1948. But there is still due to the *Journal*, for 1939–45, the balance of the £184 18s. 0d., of which £84 13s. 11d. was paid in 1947, viz. £100 4s. 1d.

